# Genome-wide identification of the AOMT gene family in wax apple and functional characterization of *SsAOMTs* to anthocyanin methylation

**DOI:** 10.3389/fpls.2023.1213642

**Published:** 2023-09-26

**Authors:** Xiuqing Wei, Liang Li, Ling Xu, Lihui Zeng, Jiahui Xu

**Affiliations:** ^1^ College of Horticulture, Fujian Agriculture and Forestry University, Fuzhou, China; ^2^ Fruit Research Institute, Fujian Academy of Agricultural Sciences, Fuzhou, Fujian, China

**Keywords:** AOMT gene family, methylation, wax apple, gene expression, anthocyanin

## Abstract

**Introduction:**

Anthocyanins are major pigments in the peels of red-series wax apple fruits, and two principal components of them, namely, the cyanin and the peonidin, are non-methoxylated and methoxylated anthocyanins, respectively. Anthocyanin O-methyltransferases (AOMTs) are an important group of enzymes that have the ability to catalyze anthocyanins methylation to promote the solubility, stability, and bioactivity of anthocyanins. Although AOMT genes have been studied in a variety of plants, the function of them in wax apple is generally not well understood.

**Methods:**

The anthocyanin composition in peels of two wax apple cultivars was determined by High Performance Liquid Chromatography Tandem Mass Spectrometry (HPLS-MS). The genome-wide analysis of the AOMT genes was performed with bioinformatics technology, and the expression patterns of different plant tissues, cultivars, fruit ripening stages, and exogenous abscisic acid (ABA) treatments were analyzed by transcriptome sequencing analysis and real-time quantitative PCR verification. An initial functional evaluation was carried out *in vitro* using recombinant the Anthocyanin O-methyltransferase Gene 5 of S. samarangense (SsAOMT5) protein.

**Results:**

Only two main compositions of anthocyanin were found in peels of two wax apple cultivars, and it was worth noting that Tub Ting Jiang cultivar contained non-methoxylated anthocyanin (Cy3G) only, whereas Daye cultivar contained both non-methoxylated and methoxylated (Pn3G) anthocyanins. A total of six SsAOMT genes were identified in the whole genome of wax apple, randomly distributing on three chromosomes. A phylogenic analysis of the protein sequences divided the SsAOMT gene family into three subgroups, and all SsAOMTs had highly conserved domains of AOMT family. In total, four types of stress- related and five types of hormone- related cis-elements were discovered in the promoter region of the *SsAOMTs*. Expression pattern analysis showed that *SsAOMT5* and *SsAOMT6* were expressed in all tissues to varying degrees; notably, the expression of *SsAOMT5* was high in the flower and fruit and significantly higher in Daye peels than those of other cultivars in the fruit ripening period. Exogenous ABA treatment significantly increased anthocyanin accumulation, but the increase of methoxylated anthocyanin content did not reach significant level compared with those without ABA treatment, whereas the expression of *SsAOMT5* upregulated under ABA treatment. We identified two homologous *SsAOMT5* genes from Daye cultivar (*DSsAOMT5*) and Tub Ting Jiang cultivar (*TSsAOMT5*); the results of functional analyses to two SsAOMT5 recombinant proteins *in vitro* demonstrated that *DSsAOMT5* showed methylation modification activity, but *TSsAOMT5* did not.

**Conclusion:**

In conclusion, SsAOMT5 was responsible for methylated anthocyanin accumulation in the peels of wax apple and played an important role in red coloration in wax apple peels.

## 1 Introduction

Plants are endowed with rich colors by abundant secondary metabolites. Flavonoids, carotenoids, and betalains are known as major secondary metabolites coloring leaves, flowers, and fruits (Du et al., 2015). Flavonoids found in virtually all vascular plants play important roles in plants, including UV and pathogens protection, pollen germination, antioxidation, and hormone transport (Provenzano et al., 2014; Niranjan et al., 2015; Gao et al., 2018). The major subclasses of flavonoids are chalcones, flavones, flavanols, proanthocyanidins, and anthocyanins (Brenda, 2001). Anthocyanins are the largest group of flavonoids and mainly responsible for a blue, red, or purple color of many plant tissues (Tanaka et al., 2009). The structural diversity of anthocyanins comes from various modification reactions, such as hydroxylation, glycosylation, acylation, and methylation.

O-methyltransferase (OMT)–mediated methylation promotes solubility, stability, and bioactivity of specific natural compounds for defense and adaptation to environment changes (Giordano et al., 2016). Because of OMTs’ role in plant secondary metabolism, the numbers and functions of OMT genes have been studied in various plants, such as arabidopsis (Niron and Türet, 2019), citrus (Liu et al., 2016; Liu et al., 2020), and opium poppy (Agarwal et al., 2019). OMT family has three major types according to their amino acid (aa) sequence and substrate variance (Noel et al., 2003). Anthocyanin OMTs (AOMTs), methylated hydroxyl groups at the 3′ and 5′ carbon in the B ring, belongs to type 1 OMTs that implicated in the methylation of flavonoids and isoflavonoids. The candidate genes involved in anthocyanin O-methylation have been investigated to better understanding the function and regulatory mechanisms. In 2011, the gene *CkmOMT2* that encoded the enzyme CkmOMT2 was revealed from purple-flowered fragrant cyclamen, and CkmOMT2 show methylation activity with anthocyanins *in vitro* (Akita et al., 2011). Subsequently, the gene *AnthOMT* was discovered from 57 candidates OMT genes in the tomato genome, in which silencing inhibited the accumulation of predominant methylated anthocyanins (Gomez Roldan et al., 2014). In addition, by comparing the two homologous genes *PsAOMT* (from purple-flowered) and *PtAOMT* (from red-flowered), Du et al. (Du et al., 2015) found that the vast difference of methylation activity to anthocyanins between PsAOMT and PtAOMT was caused by the difference in aa residue substitution at position 87.

Wax apple [*Syzygium samarangense* (BIume) Merr.et Perry] is a characteristic fruit with crisp and juicy taste and special flavor and is an economically important fruit crop in Southeast Asia such as in Malaysia, Thailand, Indonesia, and Southeast China (Supapvanich et al., 2011). In addition to low-acid taste and the aroma of roses, attractive color is also a crucial feature that makes wax apple popular in the market. The fruits are usually pink, light red, or red, and, sometimes, greenish-white or cream-colored (Khandaker et al., 2012). The deep red of peel, as a consequence of the anthocyanin accumulation, is the most popular color now. In previous studies, we found that there were methylated and non-methoxylated anthocyanins in peels of wax apple (Wei et al., 2019), but little is known about the formation mechanisms. In this study, we performed a genome-wide survey of members of the AOMT gene family in wax apple and carried out the comprehensive analysis of them, including sequence alignment, phylogenetic analyses, gene structure, protein motif, and promoter cis-element analysis. The expression profiling of AOMT genes in different tissues, cultivars, fruit ripening stages, and abscisic acid (ABA) treatment was determined by RNA- seq and real-time quantitative PCR (qRT-PCR) analysis. In addition, the potential function of AOMT genes was further clarified by an *in vitro* assay of recombinant protein.

## 2 Materials and methods

### 2.1 Plant materials and treatments

Three wax apple cultivars with different peel color were used to identify the components and contents of anthocyanin and *SsAOMT* expression pattern. Mature fruits for Tub Ting Jiang (with deep red color), Daye (with light red color), and Yangzhibai (with white color) were sampled from wax apple orchards located in Dongshan county, Zhangzhou city, Fujian Province, China (23°42′4.54″N, 117°25′48.22″E). The root, stem, leaves, flesh, flower, and ovary of Tub Ting Jiang trees were collected for the studying of gene expression patterns in various tissues. During fruit ripening period, the effect of treatment with exogenous ABA was tested on fruits of Daye cultivar, whose anthocyanin profile contains non-methylated and methylated forms. In 35 days after blossom, immature fruits on three trees were treated with 0.1 mM ABA by spraying until dripping. Treated fruits were collected at 1 h, 4 days, 7 days, and 10 days after treatment, respectively. The pericarps were separated and stored at −80°C after liquid nitrogen freeing for later analysis.

### 2.2 Anthocyanin extraction and quantitative analysis

The extraction protocol of anthocyanins was as follows: 2.5 g of pericarps were incubated with of 25 mL of MeOH-HCl (pH3) in dark for 24 h and then centrifuged at 12,000 g (RT). Supernatant was collected and dried in a vacuum (40°C) and then dissolved in 5 mL of 0.01% (v/v) HCl. All extracts were filtered through a 0.22- μm membrane before injection. Waters Acquity UPLC chromatograph with a C18 column (Luna, 5 µm, 4.6 mm × 250 mm) was used to determine the content of anthocyanins. The solvent and gradient method was as follows: solvent A, 10% aqueous formic acid; solvent B, methanol; constant gradient from 5% to 60% B within 20 min, from 60% to 100% B for 5 min, and then maintain 100% B for 5 min. The injection sling was 10 μL. The flow rate was 1 mL min^−1^. The detection was at 520 nm, and the column oven temperature was maintained at 35°C. The mass spectrometry conditions were as follows: electrospray ion source; positive ion mode; capillary voltage, 3, 500 V; nebulizer, 45 psi; dry gas: nitrogen; cone gas flow, 12 L min^−1^; dry temperature, 300°C; ion trap, scan from Mass to charge ratio (m/z) 200 to 1,300. The anthocyanins were quantified by external calibration using Cyanin-3-O-glucoside (Cy3G) and Peonidin-3-O-glucoside (Pn3G) standard (Extrasynthese, France).

### 2.3 Identification of *AOMT* genes

Eleven previously reported *AOMT* protein sequences (**Supplementary Table 1**) coming from National Center for Biotechnology Information (NCBI) database according to accession numbers were used as queries to perform Blastp searches against wax apple genome (the entire *S. samarangense* genome has been sequenced by our research group; the related paper is in preparation; Wei et al., 2023) at a cutoff *E*- value of 1 × 10^−10^. All protein sequences were checked for the presence of Pfam domains (PF01596) by Pfam (http://pfam.xfam.org) and NCBI (https://www.ncbi.nlm.nih.gov/Structure/bwrpsb/bwrpsb.cgi) database, respectively. The redundant sequences were eliminated. Then, putative genes were named as *SsAOMT1* to *SsAOMT6* based on the loci deducing from the gff-3 files of wax apple genome. The putative AOMT proteins from *Arabidopsis thaliana* TAIR10, *Solanum lycopersicum* SL3.0, *Petunia axillaris* v1.6.2, and *Vitis vinifera* 12X were identified by the similar strategy. Furthermore, the physicochemical properties were predicted by ExPASy (https://web.expasy.org/protparam/).

### 2.4 Sequence alignment and phylogenetic analyses

The full- length AOMT sequences from six plants above mentioned were aligned with Clustal W progress of MEGA6.0, and the software was used to reconstruct a phylogenetic tree too by using the maximum-likelihood test method (Akita et al., 2011), with 1,000 bootstrap replicates.

### 2.5 Gene structure, protein motif, and promoter cis-element analysis

Exon–intron structure information and conserved domain of *SsAOMT* genes were extracted from reference gene annotation data by TBtools software (Chen et al., 2020). The deduced SsAOMT protein sequences of wax apple were submitted to the online MEME (Multiple Expectation Maximization for Motif Elicitation) v.5.3.3 (https://meme-suite.org/meme/tools/meme) (Bailey et al., 2009) to analyze conserved motifs with the default parameters. Phylogenetic tree, gene structure, and conserved motif of *SsAOMT* genes were visualized using TBtools software. The aa sequences of *SsAOMTs* were aligned with Clustal W and refined manually. The 2- kb upstream sequence of each *SsAOMT* was used for cis-elements prediction in the PlantCARE database (http://bioinformatics.psb.ugent.be/webtools/plantcare/html/).

### 2.6 RNA extraction, qRT-PCR, and gene expression

The total RNA was extracted from pericarps using the Plant total RNA extraction Kit (BioTeke, Beijing, China). The integrity of RNA was assessed by 1.5% agarose gel electrophoresis, and the concentration of RNA was estimated with the Nanodrop2000C spectrophotometer (Thermo). The first strand cDNA was synthesized using HiScript^®^ III RT SuperMix for qPCR (+gDNA wiper) (Vazyme, Beijing, China) according to the manufacturer’s instructions. cDNA libraries were constructed and sequenced by Illumina NovaSeq 6000 (Illumina, USA) platform. FastQC software was used to evaluate the reads quality, and Trimmomatic (Bolger et al., 2014) was used to remove sequencing adapters and low-quality bases. We aligned clean data to the *S. samarangense* genome using HISAT2 (v2.0.5) (Kim et al., 2019) and calculated fragments per kilobase per million mapped fragment (FPKM) value using StringTie (v.1.2.3) (Garber et al., 2011). FPKM values were used to measure the expression levels of genes. The qRT-PCR mixture (10 μL) contained 1 μL of cDNA, 5 μL of 2×ChamQ Universal SYBR qPCR Master mix (Vazyme, Beijing, China), 0.4 μL of each primer, and 3.2 μL of ddH_2_O. The qRT-PCR conditions were follows: 95°C for 30 s, followed by 40 cycles of 5 s at 95°C, 34 s at 60°C, then 15 s at 95°C, 60 s at 60°C, and 15 s at 95°C. Each reaction was performed in biological triplicate. Data were analyzed using the 2^−ΔΔCt^ method. Primer sequences used in the present study are presented in **Supplementary Table 2**. The expression levels of *SsAOMTs* were normalized to that of *EF-1α* (Hao et al., 2016).

### 2.7 Characterization of recombinant AOMT

The *SsAOMT5* was amplified using the following primers AOMT5F (5′-ATGAGCATAAGCTCATCTA GCGGGG-3′) and AOMT5R (5′-CCCCGCTAGATGAGCTTATGCTCAT-3′). The sequenced cDNA of *SsAOMT5* was transferred into the pINFUSE vector, which contained the hFc tag. Human Embryonic Kidney 293 (HEK293) cells were grown in liquid medium in a rotary shaker at 130 rpm at 37°C, and, then, supernatants were harvested and centrifuged. Recombinant SsAOMT5 was purified using amylose resin column [New England Biolabs (NEB)]. Purified recombinant SsAOMT5 (2 μg) was determined in a final volume of 200 μL with 200 μM SAM and 20 μM anthocyanin substrate in 100 mM Tris-HCl (pH 7.5), containing 1 mM MgCl_2_ and 14 mM β-mercaptoethanol. The boiled recombinant SsAOMT5s were used as the negative controls. The reaction mixture was incubated at 35°C for 30 min and terminated with 800 mL of methanol containing 0.2% formic acid, followed by centrifugation at 12,000 rpm for 10 min. The supernatant was taken, passed through a 0.22- µm microporous membrane and detected by HPLC.

### 2.8 Statistical analysis

SPSS 26.0 statistical software (SPSS, USA) was used for data analysis. Data were presented as means ± SD of three independent replicates. Independent sample T- test was used for data analysis of two groups, and the one-way analysis of variance (ANOVA) was used for analysis of >2 groups.

## 3 Results

### 3.1 Identification of the anthocyanin compounds in peels of two wax apple cultivars

**Figure 1:**
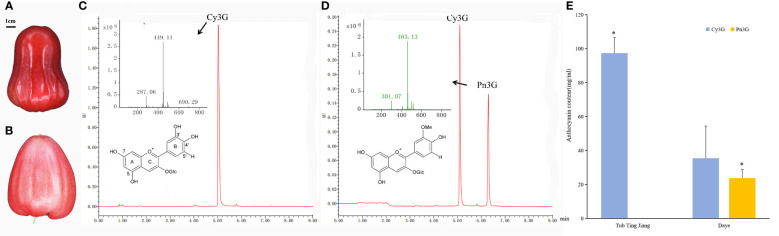
The anthocyanin components and contents in peels of two wax apple cultivars. **(A, B)** The fruit of Tub Ting Jiang **(A)** and Daye **(B)** cultivars, respectively. **(C, D)** HPLC-MS chromatograms at 520 nm of peel extracts of Tub Ting Jiang **(C)** and Daye **(D)**, and the molecular structure of cyanin-3-O-glucoside (Cy3G) and Peonidin-3-O-glucoside (Pn3G). **(E)** The contents of anthocyanin component in peels of two wax apple cultivars. The differences analysis of the same anthocyanin component content between two cultivars was performed. Cy3G, cyanin-3-O-glucoside; Pn3G, peonidin-3-O-glucoside; * Significant (p < 0.05); Bar = SD.

Chromatograms at 520 nm of peel extracts from Tub and Daye cultivars were presented in **Figure 1**. Two main individual anthocyanin compounds, namely, Cy3G (m/z 449 [M]^+^; m/z 287 [M]^+^) and Pn3G (m/z 463 [M]^+^; m/z 301 [M]^+^), were identified in wax apple peels using HPLC-MS (Johnson et al., 2015). Identified Cy3G in wax apple was consistent with previous research (Reynertson et al., 2008). It should be noted that Cy3G was detected in both two cultivars, and Pn3G was only found in Daye cultivar. The Cy3G content of Tub cultivar was 97.40 mg mL^−1^; it was extremely significantly higher than that of Daye cultivar (35.33 mg mL^−1^). The Pn3G content (23.78 mg mL^−1^) was slightly lower than that of Cy3G in Daye cultivar and amounted to 40.2% of the total anthocyanin. It was known that Pn3G was a methylated anthocyanin by methylation of 3′- hydroxyl groups at the B-ring of Cy3G. Therefore, Daye cultivar contained both non-methylated and methylated anthocyanins, whereas Tub cultivar contained non-methylated anthocyanins only. The above results indicated that differences of methylation modification existed between two cultivars in the anthocyanin biosynthesis pathway.

### 3.2 Identification and analysis of phylogenetic, structure, motif, and cis-elements of SsAOMT genes in wax apple

#### 3.2.1 Identification and phylogenetic analysis of SsAOMT genes

**Figure 2:**
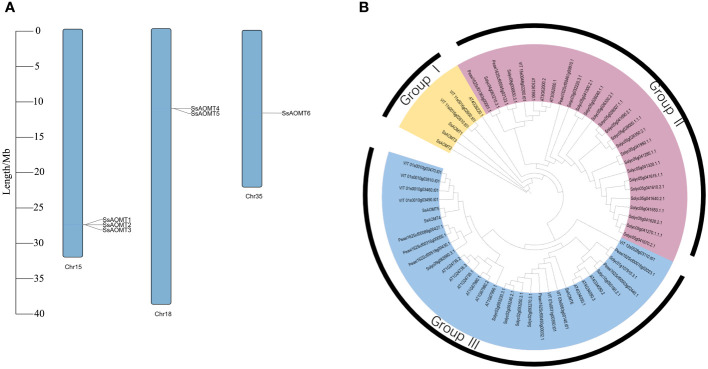
Chromosomal distribution and phylogenetic tree. **(A)** The distribution of *SsAOMTs* in chromosomes of wax apple. **(B)** Phylogenetic tree of 65 AOMT proteins from *S. samarangense*, *A. thaliana*, *S. lycopersicum*, *P. axillaris*, and *V.vinifera*. The colored arcs indicated different groups. Ss, *S. samarangense*; AT, *A. thaliana*; Solyc, *S. lycopersicum*; Peaxi, *P. axillaris*; VIT, *V.vinifera*. Chr15, Chr 18 and Chr 35 indicated the Chromosome 15, 18 and 35, respectively.

On the basis of previous reports, we screened 11 aa sequences of AOMT conserved domain of various plants as queries to obtain AOMT genes from wax apple genome. Six non-redundant candidate AOMT genes were identified and named as *SsAOMT1* to *SsAOMT6* according to their position on the chromosome (**Table 1**, **Figure 2A**). The putative AOMT proteins in other plants genome were also identified by the similar strategy, including *A. thaliana*, *S. lycopersicum*, *P. axillaris*, and *V.vinifera*. There were 13, 27, 9, and 10 *AOMT* genes identifying from *A. thaliana*, *S. lycopersicum*, *P. axillaris* and *V.vinifera*, respectively (**Supplementary Table 3**).

**Table 1:** The basic information of SsAOMT gene family.

Gene name	Gene length (bp)	CDS length (bp)	Exon numbers	Amino acids length (aa)	Theoretical Mw (kDa)	Theoretical PI	Instability index (Inindex)	Chromosome location		
*SsAOMT1*	992	308	5	102	11.39	5.8	33.65	Chr15	27651044	27652035
*SsAOMT2*	1,970	717	5	238	26.79	5.05	33.54	Chr15	27667921	27669890
*SsAOMT3*	1,807	717	6	239	26.79	5.17	30.89	Chr15	27680727	27682533
*SsAOMT4*	3,319	708	5	236	26.5	5.24	30.59	Chr18	11272004	11275322
*SsAOMT5*	1,545	648	4	216	23.92	4.81	37.44	Chr18	11282303	11283847
*SsAOMT6*	2,095	741	5	247	27.69	5.61	33.84	Chr35	11721300	11723394

The six *SsAOMT* genes located unevenly on three chromosomes, three genes on the chromosome 15, two genes on the chromosome 18, and only one gene was distributed in chromosome 35. Sequence feature analysis of *AOMT* genes suggested that the gene length ranged from 992 to 3,319 bp, whereas deduced aa sequence lengths of *SsAOMTs* varied from 102 (*SsAOMT1*) to 239 aa (*SsAOMT3*), with an average of 213 aa. Most of *SsAOMTs* contained five exons, except for *SsAOMT3* that had six and for *SsAOMT5* that had 4 exons. They had the lowest Molecular Weight (MW) of 11.39 kDa (*SsAOMT1*) and the highest MW of 27.69 kDa (*SsAOMT6*). The Isoelectric Point (PI)-value ranged from 4.81 for *SsAOMT5* to 5.80 for *SsAOMT1*. The PI-value indicated that all *SsAOMT* proteins were acidic. The instability index showed that all of *SsAOMT* proteins were stable (Index < 40). The phylogenetic tree with total 65 *AOMT* genes from five species was constructed on the basis of their protein sequences. All these *AOMT* sequences could be classified into three main groups (namely, group I, group II, and group III; **Figure 2B**) including 6, 27, and 32 members, respectively. Six *SsAOMTs* were mainly distributed in group I (*SsAOMT1*, *SsAOMT2*, and *SsAOMT3*) and group III (*SsAOMT4*, *SsAOMT5*, and *SsAOMT6*), whereas none *SsAOMT* belonged to group II.

#### 3.2.2 Structure analysis and motif composition of SsAOMT genes

**Figure 3:**
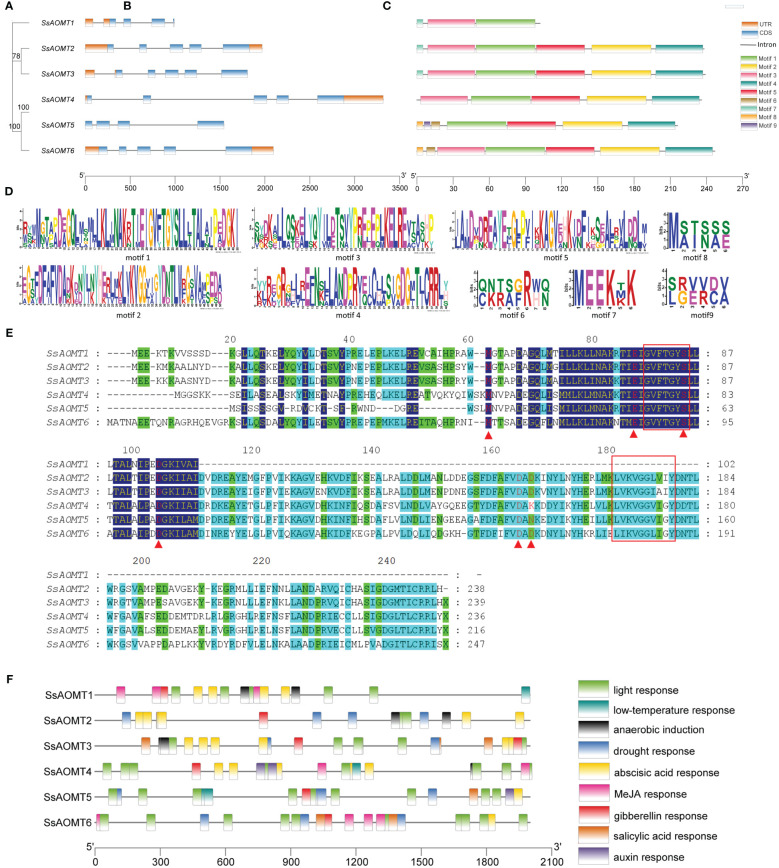
Structure analysis of *SsAOMT* genes. **(A)** Phylogenetic tree, **(B)** gene structure, **(C)** conserved motif distribution, **(D)** nine conserved motifs, **(E)** sequence alignments, and **(F)** cis-elements in the promoter sequences (2 kb upstream). Node values beside branches were bootstrap-supported values generated from 1,000 replicated in **(A)**. Red box remarked SAM-binding domains; red triangle under the column remarked the putative SAM-binding residues in **(E)**.

The gene structure is an important basis for studying polygenic families; therefore, we analyzed the structural diversity of *SsAOMTs*. The details of exons and introns within each of the *SsAOMT* genes are shown in **Figure 3B**. All the *SsAOMT* genes contained four introns except *SsAOMT3* and *SsAOMT5*. The longest intron was found in *SsAOMT4*, whereas the second and third longest introns appeared in *SsAOMT5* and *SsAOMT6*, which were in the same subclade with *SsAOMT4* (**Figure 3A**). Nevertheless, the number of exons in all *SsAOMT* genes stably ranged from 4 to 5, indicating that gene structure may be relatively conserved among *SsAOMT* family members.

A total of nine distinct motifs (motif 1 to motif 9) were detected among *SsAOMT* proteins, and the length of protein sequences to these motifs varied from 6 to 50 aa (**Figure 3C**). The majority of *SsAOMT* proteins possessed more than four motifs, except *SsAOMT1* protein showed only three motifs, and we noticed that motif 1 was found on all *SsAOMT* proteins. Furthermore, we made a multiple alignment analysis of six SsAOMT sequences. The GVXTGYS and LVXXGGXI sequences, which were considered as S-AdenosvlmeIhionine (SAM)-binding domains (Joshi and Chiang, 1998), were discovered in motif 1 and motif 2 (**Figures 3D, E**). Met-63, Glu-87, Ser-95, Asp-106, Asp-165, and Asp-167 were conserved in SsAOMTs (**Figure 3E**) and were putative SAM-binding residues with reference to the three-dimensional models of PsAOMT and PtAOMT (Du et al., 2015). The deduced protein sequences of SsAOMTs were aligned with 11 previously reported AOMTs (**Supplementary Table 1**) to show the conserved domains which was consistent with the previous description (**Supplementary Figure 1**).

#### 3.2.3 Analysis of cis-elements in *SsAOMT* genes

Cis-acting elements play key roles in transcriptional control that is an important method of regulating gene expression. To understand the potential regulatory mechanisms of *SsAOMT*, the upstream 2- kb sequences from the translation start sites of *SsAOMTs* were used to predict the cis-elements by the databases of PlantCARE. A number of cis-elements were found; other than a high number of promoter/enhancer elements (TATA-box, CAAT-box), there were many other elements responding to environmental stresses and hormones (**Supplementary Table 4**). In total, four types of stress- related and five types of hormone- related cis-elements in the promoters are shown in **Figure 3F**. As can be seen, light ABA- and gibberellin- responsive elements were distributed randomly on all *SsAOMTs*, and some elements were arranged in cluster in certain promoters, such as light -responsive elements (G-box and I-box) in *SsAOMT4* and *SsAOMT6*. However, other six types of cis-elements were absent in some *SsAOMTs*, for example, MeJA -responsive element (CGTCA-motif) was absent in *SsAOMT2* and *SsAOMT5*.

### 3.3 Expression patterns of *SsAOMT* genes

#### 3.3.1 Expression profiles of *SsAOMT* genes in different plant tissues

Gene expression pattern was a useful tool to study its function; therefore, the RNA-seq data of six organs from root, stem, leaf, flower, fruit, and ovary were used to assess the expressions pattern of six *SsAOMTs*. As seen from **Figure 5A**, *SsAOMT*5 and *SsAOMT*6 were found to be expressed in all tissues, *SsAOMT*5 had highest expression in flower and higher expression in fruit and stem, and *SsAOMT*6 showed higher expression in leaf and high expression in flower and stem. The other *SsAOMTs* were almost not expressed in all tissues except *SsAOMT1* in flower and *SsAOMT4* in stem.

#### 3.32 Expression patterns of *SsAOMT* genes in pericarps of different cultivars

To further understand the physiological roles of *SsAOMTs* in anthocyanin methylation, we observed the expression patterns of *SsAOMTs*. As the data shown in **Figure 5B**, expression patterns of *SsAOMTs* for three cultivars were different; *SsAOMT1*, *SsAOMT2*, and *SsAOMT3* had nearly no expression in peels; conversely, the other genes’ expressions were observed in all cultivars with different degrees. On the basis of difference analysis, we found that the difference of expressions for *SsAOMT4* and *SsAOMT5* among three cultivars was significant. Moreover, the expression of *SsAOMT6* in Tub Ting Jiang cultivar was extremely significantly lower than that in Daye and Yangzhibai cultivars, but there was no significant difference of expression between Daye and Yangzhibai cultivars. Notably, *SsAOMT5* expressed highest in Daye cultivar was probably correlated with methylated anthocyanins biosynthesis.

#### 3.3.3 Expression patterns of *SsAOMT* genes during fruit ripening stages

The relative expression levels of *SsAOMTs* were investigated during fruit ripening development of wax apple. The qRT-PCR analysis results were shown in **Figure 5C**. Because no expression of *SsAOMT1*, *SsAOMT2*, and *SsAOMT3* was detected, they were not shown in the figure. *SsAOMT4*, *SsAOMT5*, and *SsAOMT6* were expressed to varying degrees, and *SsAOMT5* showed a much higher expression than *SsAOMT4* and *SsAOMT6* throughout the ripening development. The expression patterns of three *SsAOMTs* could largely be classified into two patterns. Pattern 1 included two genes (*SsAOMT4* and *SsAOMT5*) that was upregulated with fruit developmental process overall. Pattern 2 comprised one gene (*SsAOMT6*) that showed downregulated expression in Tub Ting Jiang cultivar and fluctuating expression in Daye cultivar (**Figure 5C**). In addition, the *SsAOMT5* expression of Daye cultivar was 10 times higher than of Tub Ting Jiang cultivar at 50 days after full bloom (T5 stage); this was likely associated with the accumulation of methylated anthocyanins in Daye fruit pericarps.

#### 3.3.4 Effects of anthocyanin content and *SsAOMT* gene expression under exogenous ABA treatments

**Figure 4:**
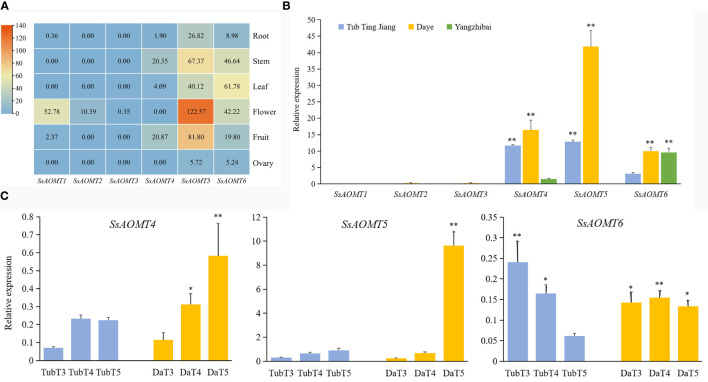
Expression Patterns of *SsAOMT* genes in wax apple. **(A)** Tissue expression profile. **(B)** Three cultivars expression profile. **(C)** Real-time relative expressions of three *SsAOMTs* in two wax apple cultivars during fruit ripening. Tub, Tub Ting Jiang cultivar; Da, Daye cultivar; T3-T5, fruit ripening stages when 30, 40, and 50 days after full bloom, respectively. * Significant (p < 0.05). ** Extremely significant (p < 0.01); Bar = SD.

**Figure 5:**
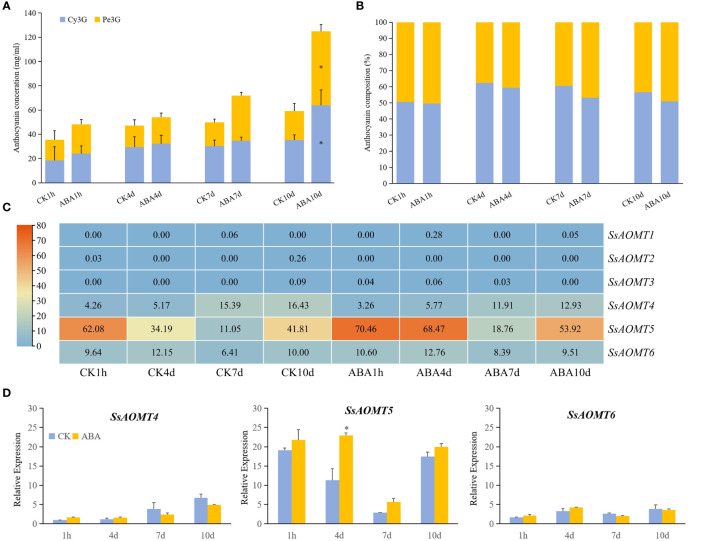
The effect of anthocyanins and *SsAOMT* expression in Daye peels with exogenous ABA treatment. **(A)** Content of anthocyanins. **(B)** Composition of Cy3G and Pn3G. **(C)** Expression profile of SsAOMTs. **(D)** Real-time relative expressions of three *SsAOMTs*. Cy3G, cyanidin 3-O-glucoside; Pe3G, peonidin 3-O-glucoside. * Significant (p < 0.05); Bar = SD.

The results of predicted cis-elements shown that all of *SsAOMT* genes contained ABA response element (**Figure 4**), and ABA has been shown to promote the accumulation of anthocyanin in many plant species (Sun et al., 2019; Han et al., 2021). To verify the predicted results, Daye fruits were sprayed ABA aqueous solution (100 μmol g^−1^) during fruit coloring with water being taken as control. The results shown that the total content of anthocyanin was significantly increased at 10 days after treated, and the concentration of Cy3G and Pe3G in the peels rose from 35.33 mg g^−1^ to 63.79 mg g^−1^ and from 23.78 mg g^−1^ to 61.17 mg g^−1^, respectively (**Figure 5A**). To get more direct impression about the effect of ABA to anthocyanin methylate, the composition of Cy3G and Pe3G in peels was shown in **Figure 5B**. The percentage of Pe3G, a methylated anthocyanin, increased after 7 and 10 days of treatment, although it did not reach any significant level compared with control.

The transcriptome data showed that only the change of *SsAOMT5* expression was more obvious under ABA treatment, whereas the expression of *SsAOMT4* and *SsAOMT6* did not change significantly, and *SsAOMT1*, *SsAOMT2*, and *SsAOMT3* were nearly not expressed (**Figure 5C**). The expression of three candidate genes was further validated using qRT-PCR, we found that only *SsAOMT5* upregulated in different extent after ABA treatment, especially the degree of upregulation of 4 days after treatment was highest (**Figure 5D**). In general, the expression pattern of *SsAOMT5* accorded with the increasing of Pe3G percentage after ABA treatment.

### 3.4 Functional validation *in vitro* of recombinant SsAOMT5

**Figure 6:**
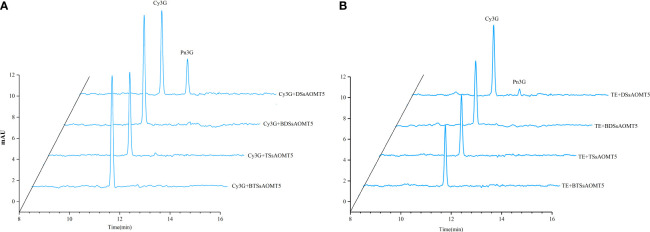
Analysis of SsAOMT5 *in vitro* reaction products by HPLC. Standards Cy3G and TE as substrates for recombinant DSsAOMT5 and TSsAOMT5, respectively. BDSsAOMT5 and BTSsAOMT5 denatured by boiling for 5min were used as negative control. DSsAOMT5 and TSsAOMT5 were recombinant proteins expressed and purified from Daye and Tub Ting Jiang cultivars, respectively. **(A)**, the liquid chromatogram with Cy3G as substrate; **(B)**, the liquid chromatogram with TE as substrate. Cy3G, cyanidin 3-O-glucoside; Pe3G, peonidin 3-O-glucoside; TE, the peel extracts of Tub Ting Jiang cultivar.

To confirm our hypothesis that *SsAOMT5* correlated closely with methylated anthocyanins, we cloned and expressed recombinant SsAOMT5 from Daye and Tub Ting Jiang and were designated DSsAOMT5 and TSsAOMT5, respectively. Two genes with Open Reading Frames (ORFs) of 690 Base Pair (bp) both contained the SAM-binding domains and encoded two 230 aa proteins and calculated molecular mass of 25.9 kDa and 25.8 kDa, respectively (**Supplementary Figures 2–4**). Through sequence alignment, we found that a total of 4 aa sites (positions 127, 185, 210, and 217) were different between two proteins (**Supplementary Figure 3**). Both recombinant DSsAOMT5 and TSsAOMT5 had molecular weights of approximately 49 kDa. The biological activities of purified recombinant DSsAOMT5 and TSsAOMT5 were measured using the Cy3G standard and the peel extract of Tub Ting Jiang cultivar as substrates. The results shown that DSsAOMT5 could use both two substrates as methoxyl accepters, and it methylated the 3′-hydroxyl group of the B-ring of Cy3G into Pn3G; however, when Cy3G standard was used as the substrate, the efficiency was higher than that when the peel extract as the substrate (**Figures 6A, B**). In contrast, we could not detect the methylated products of TSsAOMT5 reaction system, which suggested that TSsAOMT5 did not exhibit any methylation activity with both the Cy3G standard and the peels’ extract of Tub Ting Jiang cultivar (**Figures 6A, B**).

## 4 Discussion

The wax apple skin color was mainly attributed to the accumulation of anthocyanins; our study showed that the main anthocyanins in wax apple were cyanidin (methylated anthocyanin) and peonidin (non-methylated anthocyanin), but the ratios of them were variable among the cultivars (Wei et al., 2019). We found that Tub Ting Jiang cultivar accumulated cyanidin in the skin only; however, nothing is known about the mechanisms underlying this phenomenon. To our knowledge, AOMT was responsible for the methylation of anthocyanins and, in turn, improved the stability of anthocyanin and contributed to the red or purple coloration of plants’ flowers and peels (Du et al., 2015) It has been identified a few *AOMTs*, such as grape *VvAOMT* (Fournier-Level et al, 2011; Giordano et al., 2016), cyclamen *CkmOMT2* (Akita et al., 2011), tomato *AnthOMT* (Gomez et al., 2014), paeonia *PsAOMT* and *PtAOMT* (Du et al., 2015), and pomegranate *PgAOMTs* (Zhang et al., 2021), respectively. Therefore, we explored AOMT genes in genome-wide data of *S. samarangense* using genome mining. In this study, we identified six *SsAOMTs* putative genes that were unevenly distributed on three chromosomes (15, 18, and 35). Tandem duplication and segment duplication as a small- scale genomic duplication significantly contribute to gene family expansion (Liu et al., 2017; Run et al., 2021). Two tandem- duplication events and no segment duplication were observed in six *SsAOMTs* (**Figure 2A**); therefore, in present study, tandem- duplication events might play an important role in the evolution of the *SsAOMT* genes in wax apple. Gene structural is the basis of the studying for multigene families and plays a key role in the evolution of them (Ji et al., 2019); our results showed that members of the SsAOMT protein families displayed sequence homology and structure conservation, and all *SsAOMTs* contained SAM-binding domains by motif analysis. On the basis of the phylogenetic tree and homology analysis, a total of 65 genes from five species were classified into three groups, and six *SsAOMTs* were distributed to two of these groups (**Figure 2**). The two groups of *SsAOMTs* showed different expression patterns. *SsAOMT1*, *SsAOMT2*, and *SsAOMT3* were in the same group (group I) and not expressed in all tissues except that *SsAOMT1* and *SsAOMT2* scantly expressed in flowers (**Figure 5A**); this might suggest that they were pseudogenes. Similar results were found in pomegranate (Run et al., 2021); this was most likely attributed to rapid birth and death of genes during evolution (Masatoshi and Alejandro, 2005). *SsAOMT4*, *SsAOMT5*, and *SsAOMT6* belonged to group III; *SsAOMT5* and *SsAOMT6* were expressed in various organs including root, stem, leaf, flower, and fruit; notably, the high expression level of *SsAOMT5* in the flower and fruit indicated that it may be linked to anthocyanin methylation of wax apple. In addition, the number of *AOMTs* in wax apple was lower than that of pomegranate (10) throughout the whole genome wide. The type of anthocyanins in wax apple skin was less than that of pomegranate peel, which includes cyanidin, delphinidin, and pelargonidin (Zhao et al., 2015); we therefore speculate that the requirements for transcripitional regulation to anthocyanin methylation of wax apple were less than pomegranate during evolution.

The expression pattern of *SsAOMTs* provides hints to further investigation of their biological function. Therefore, in addition to *SsAOMT* expression in different plant tissues, we also further studied the expression patterns of *SsAOMTs* among different cultivars and different fruit developmental stages based on RNA- seq and qRT-PCR technology. Results of previous studies have shown that AOMTs can specifically methylate hydroxyls in the 3′ and 5′ positions (Provenzano et al., 2014; Du et al., 2015), for example, the allelic variants of VvAOMT2 of grape caused changes in substrate-specific catalytic efficiency (Fournier-Level et al., 2011). From six *SsAOMT* gene expression patterns of three wax apple cultivars, it is known that the expression of *SsAOMT5* in Daye cultivar was significantly higher than that of the other two cultivars; combined with that, the methylated anthocyanin (Pe3G) was discovered in the skin of Daye fruits only, so we speculated that *SsAOMT5* was a major gene affecting anthocyanin methylation of wax apple. Moreover, low or no expression of *SsAOMT1*, *SsAOMT2*, and *SsAOMT3* in the skin to all three wax apple cultivars may once again prove our supposition that they were pseudogenes (Run et al., 2021). To further understand *SsAOMT* expressions during fruit ripening period, two wax apple cultivars with significant differences were used for the studies. During fruit ripening period, *SsAOMT5* was upregulated continuously in both Tub and Daye cultivars and got the peak at 50 days after blossom (T5); especially, the expression of *SsAOMT5* in Daye cultivar increased sharply and was extremely significantly higher than that in Tub cultivar at T5 stage. This was different to the results of pomegranate, in which *PgOMT04* and *PgOMT09* in peel were always kept with a high expression throughout fruit development (Zhang et al., 2021), and also different to the finding that *NmAMT* in petals of *N. menziesii* significantly expressed only at the early stages of flower development (Okitsu et al., 2018). This discrepancy was most likely related to species. Together, we presumed that the high expression level of SsAOMT5 was contributed to the accumulation of Pe3G, which was a kind of methylated anthocyanins in the skin of Daye cultivar.

Four aa differences (Asp-127, Glu-185, Val-210, and Val-217) were noted in the *SsAOMT5* between two cultivars of wax apple by cloning and sequencing. The results of functional validation *in vitro* for recombinant SsAOMT5 revealed differences in the catalytic properties of DSsAOMT5 and TSsAOMT5, and TSsAOMT5 exhibited no cyanidin methylation activity. Previous studies have revealed that the change of key aa residues could either promote or inhibit enzyme activity (Forman et al., 2018; Zorn et al, 2018). In *Paeonia* spp., the aa sequences of PsAOMT (from purple-flowered) and PtAOMT (from red-flowered) also differed in four residues; the single aa residue (Leu-87) was confirmed as a critically important residue affecting the activity of PsAOMT by site-directed mutagenesis (Du et al., 2015). However, Leu-87 was far from the putative substrate-binding pocket. The aa residues appearing to be involved in the ligand binding pocket or activity site of the enzyme usually significantly affect the catalytic activity of enzyme. Moreover, several residues upstream and downstream of the binding pocket and specificity site also influence enzyme activity by a conformational change of the substrate-binding region. When the aa residues upstream and downstream from the binding motif and specificity motif of MdOSC1 were mutated, Yu et al. (2020) found that, among 40 mutants, five exhibited higher enzyme activity, three displayed reduced enzyme activity, and others lost enzyme activity. Xue et al. (2018) noted that the key aa residues #732 of Oxidosqualene Cyclases (OSCs) in *Oryza* might responsible for transformation of C-B-C and C-sC-C conformations without the formation of the E ring and, in turn, affected the catalytic efficiency and function of OSC. Compared with the AOMT protein sequence of the other eight species, although the conserved domains were prominent, four different aa residues found in SsAOMT5 were not well conserved among species (**Supplementary Figure 1**). More related studies such as site-directed mutagenesis and X-crystal structures are needed to be initiated to gain the key aa residues and further insights into the catalytic mechanisms of SsAOMT5.

The cis-elements in promoter region can regulate gene expression (Carlos and John, 2014). For the thorough understanding of the regulation of *SsAOMT* gene expression, the cis-regulatory elements in the *SsAOMT* promoter regions were analyzed. Three main types of stress cis-regulatory elements were discovered, including phytohormones, photoresponsive, and environmental stress elements (**Figure 4**). All *SsAOMT* genes contained abscisic acid response element. Studies have shown that exogenous ABA treatment increased the anthocyanin content in plant species (Wei et al., 2014; Han et al., 2021). Koyama et al. (2018) found that the concentrations of peonidin-3-glucoside and malvidin-3-glucoside, which were the methylated anthocyanins, increased with increasing of total anthocyanin content in grape fruits, when they used exogenous ABA to treat a new grape lacking of red color when grown in subtropical areas. Our results were in accordance with the above findings. The total anthocyanin content of wax apple significantly increased after 10 days of exogenous ABA treatment, and the percentage of Pe3G in the total anthocyanin slightly increased. The expression of *SsAOMT5* was upregulated in varying degree at different times after exogenous ABA treatment. This might imply a possible link between application of exogenous ABA and the accumulation of methylated anthocyanins.

## 5 Conclusion

In summary, we found that the major anthocyanins in wax apple were cyanin and peonidin, and the major components content varied between the cultivars. A total of six *SsAOMT* genes were identified by genome-wide exploration, and they were divided into three subgroups by phylogenetic analysis. Furthermore, the gene structures, functional motifs, and cis-elements were identified; the results showed that *SsAOMTs* were highly conserved. In addition, comprehensive analyses of the expression profiles revealed that *SsAOMT5* may have an important role in anthocyanin methylation of wax apple. In an *in vitro* assay, we further demonstrated that recombinant protein DSsAOMT5 had catalytic activity in the methylation of anthocyanin.

## Data availability statement

The data presented in the study are deposited in the CNCB repository (https://ngdc.cncb.ac.cn/), accession numbers CRA010949 and CRA010957.

## Author contributions

XW performed the experiments and wrote the manuscript. LL conducted data analysis and assisted with the manuscript. LX assisted with samples collection and experiments. LZ guided the subject design and the paper writing. JX provided experiments advice and funding. All authors contributed to the article and approved the submitted version.
