# Supplementary material for: Genome-wide identification of the AOMT gene family in wax apple and functional characterization of SsAOMTs to anthocyanin methylation
Source: Front Plant Sci. 2023 Sep 26;14:1213642. doi: 10.3389/fpls.2023.1213642 (PMC10562569; doi:10.3389/fpls.2023.1213642)
Supplement: Supplementary file 1 [file DataSheet_1.zip › Datasheet 1/Supplementary Tables 1-3 and Figures 1-4.doc]

**Supplemental Table 1. Biochemically characterized flavonoid OMTs**

| Name | Origin | Accession numb | Reference |
| --- | --- | --- | --- |
| CkmOMT2 | *cyclamen* | BAK74804.1 | Akita et al.,2011 |
| NmAMT6 | *Nemophila menziesii* Hook. and Arn. | LC328823 | Okitsu et al., 2018 |
| PhMF1 | *Petunia hybrida* | KJ676512 | Provenzano et al.,2014 |
| PhMF2 | *Petunia hybrida* | KJ676514 | Provenzano et al.,2014 |
| PhMT2 | *Petunia hybrida* | KJ676515 | Provenzano et al.,2014 |
| AnthOMT | *Solanum lycopersicum* | KJ850478 | Gomez et al.,2014 |
| VvFAOMT | *Vitis vinifera* | EC932040 | Lucker et al.,2010 |
| VvAOMT | *Vitis vinifera* | C7AE94.1 | Sun et al.,2018 |
| PsAOMT | *Paeonia* | FE529149 | Du et al.,2015 |
| McPFOMT | *ice plant* | AY145521 | Du et al.,2015 |
| GmAOMT | *Glycine max* | ADX43927 | Kovinich et al., 2011 |

**Supplemental Table 2. Primers used in this research work**

| Primer name | Sequences (5'→3') |
| --- | --- |
| AOMT1F | ATGGAAGAGAAAACGAAAGTAGTAT |
| AOMT1R | ATACTACTTTCGTTTTCTCTTCCAT |
| AOMT2F | ATGGAAGAGAAAATGAAAGCAGCAT |
| AOMT2R | ATGCTGCTTTCATTTTCTCTTCCAT |
| AOMT3F | ATGGAAGAGAAAAAGAAAGCAGCAT |
| AOMT3R | ATGCTGCTTTCTTTTTCTCTTCCAT |
| AOMT4F | ATGGGAGGCTCCAAGAAGAGCGAGA |
| AOMT4R | TCTCGCTCTTCTTGGAGCCTCCCAT |
| AOMT5F | ATGAGCATAAGCTCATCTAGCGGGG |
| AOMT5R | CCCCGCTAGATGAGCTTATGCTCAT |
| AOMT6F | ATGGCCACCAATGCAGAGGAGACCC |
| AOMT6R | GGGTCTCCTCTGCATTGGTGGCCAT |
| EF-1αF | AAGCCACTCCGCCTTCCCTTAC |
| EF-1αR | TGACAACCATACCAGGCTTGAGGAT |

**Supplemental Table 3. List of AMOT gene family that were used to construct a phylogenetic tree.**

| Origin | Gene ID | |
| --- | --- | --- |
| Arabidopsis thaliana TAIR10 | AT4G34050.3 | AT3G62000.1 |
|  | AT1G24735.1 | AT4G34050.2 |
|  | AT4G26220.1 | AT3G62000.2 |
|  | AT1G24735.2 | AT1G67980.1 |
|  | AT1G24735.3 | AT1G67990.1 |
|  | AT1G67980.2 | AT4G34050.1 |
|  | AT3G61990.1 |  |
| Solanum lycopersicum SL3.0 | Solyc03g032220.3.1 | Solyc05g026350.2.1 |
|  | Solyc04g063210.3.1 | Solyc08g006830.3.1 |
|  | Solyc10g050160.2.1 | Solyc05g041670.2.1 |
|  | Solyc02g093250.3.1 | Solyc05g041615.1.1 |
|  | Solyc02g093270.3.1 | Solyc05g041650.1.1 |
|  | Solyc01g107910.3.1 | Solyc05g041610.2.1 |
|  | Solyc02g093230.3.1 | Solyc05g026040.1.1 |
|  | Solyc09g082660.3.1 | Solyc05g041300.2.1 |
|  | Solyc05g041690.2.1 | Solyc05g026007.1.1 |
|  | Solyc05g041320.1.1 | Solyc05g041270.1.1.1 |
|  | Solyc05g041950.1.1 | Solyc02g093240.2.1 |
|  | Solyc05g041620.2.1 | Solyc05g026000.1.1.1 |
|  | Solyc05g026330.2.1 | Solyc05g041285.1.1 |
|  | Solyc05g041640.2.1 |  |
| Petunia axillaris v1.6.2 | Peaxi162Scf01363g00001.1 | Peaxi162Scf00003g02440.1 |
|  | Peaxi162Scf00016g02023.1 | Peaxi162Scf00461g00810.1 |
|  | Peaxi162Scf00450g00032.1 | Peaxi162Scf00045g02123.1 |
|  | Peaxi162Scf00518g00430.1 | Peaxi162Scf00089g00427.1 |
|  | Peaxi162Scf00316g00055.1 |  |
| Vitis vinifera 12X | VIT_11s0016g02600.t01 | VIT_01s0010g03490.t01 |
|  | VIT_11s0016g02610.t01 | VIT_01s0010g03510.t01 |
|  | VIT_12s0028g03110.t01 | VIT_01s0010g03460.t01 |
|  | VIT_03s0063g00140.t01 | VIT_01s0010g03470.t01 |
|  | VIT_07s0031g00350.t01 | VIT_15s0048g02200.t01 |

**Figure S1. Multiple sequence alignment of OMT protein sequences of nine species.**

**Red box remarked SAM-binding domains; eleven OMTs proteins of eight species showed in the Supplemental Table 1. The red triangles indicate the sites of four different amino acid residues found in SsAOMT5.**

**Tub Ting Jiang-*SsAOMT5:***

ATGAAGATCCTCGCAAGCGAAGCTCTCTCGAAGTACATATTGGAAACAAATGCGTATCCGAGAGAGCACGAGCAGCTGAAAGAACTCAGAGAGGTCACTGTTCAGAAGTACCAGTACTGGAGTTTGATGAATGTGCCGGCTGACGAGGGGCAACTGATCTCCATGATTTTGAAGCTCATGAATGCAAAGAGGACGATAGAGATCGGAGTCTTCACCGGCTATTCTCTCCTCACCACCGCGCTTGCACTTCCGGCCGACGGCAAGATATTGGCGATGGATCCGGACAGGGAGGCCTATGAAACGGGCCTGCCATTTATCAAGAAAGCCGGAGTCGACCATAAGATCAACTTCATCCATTCAGATGCTTTCTTGGTTCTAAACGACCTCATCGAAAATGGTGAAGAAGCGGGGGCCTTTGATTTTGCCTTTGTGGACGCCAACAAGGAGGACTACATCAAGTACCACGAGATCCTGCTCAAGCTGGTGAAGGTTGGAGGAGTGATCGGGTACGATAACACCCTATGGTTCGGGGCAGTCGCACTCTCTGAAGACGACGAGATGGCGGAGTATCTAAGGGTCGGGAGGGGCCACCTCAGGGAGCTGAATAGCTTCCTGGCGAATGATCCGCGCGTCGAGTGCTGTCTTCTTTCCGTCGGCGACGGACTCACGCTCTGTAGGCGGCTCTACAAG

**Daye-*SsAOMT5:***

ATGAAGATCCTCGCAAGCGAAGCTCTCTCGAAGTACATATTGGAAACAAATGCGTATCCGAGAGAGCACGAGCAGCTGAAAGAACTCAGAGAGGTCACTGTTCAGAAGTACCAGTACTGGAGTTTGATGAATGTGCCGGCTGACGAGGGGCAACTGATCTCCATGATTTTGAAGCTCATGAATGCAAAAAGGACGATAGAGATCGGAGTCTTCACCGGCTATTCTCTCCTCACCACCGCGCTTGCACTTCCGGCCGACGGCAAGATATTGGCGATGGATCCGGATAGGGAGGCCTATGAAACAGGCCTGCCATTTATCAAGAAAGCCGGAGTCGACCATAAGATCAACTTCATCCATTCAGATGCTTTCTTGGTTCTAAACAACCTCATCGAAAATGGCGAAGAAGCGGGGGCCTTTGATTTTGCCTTTGTGGACGCCAACAAGGAGGACTACATTAAGTACCACGAGATCCTGCTCAAGCTGGTGAAGGTCGGAGGAGTGATCGGGTACGATAACACCCTGTGGTTCGGGGCAGTCGCGCTCTCTGAAGACGACAAGATGGCGGAGTATCTAAGGGTCGGGAGGGGCCACCTCAGGGAGCTGAATAGCTTCCTGGCAAATGATCCGCGCACCGAGTGCTGTCTTCTTTCCATCGGAGATGGACTCACGCTCTGTAGGCGGCTCTACAAG

**Figure S2. The amino acid sequence of the cloned genes of *SsAOMT5.***

**Figure S3. Protein sequence alignment of *TSsAOMT* and *DSsAOMT* genes, with identical amino acids in dark blue and similar amino acids in light blue.**

**Figure S4. SDS-PAGE of recombinant SsAOMTs. Lane M: protein size markers; Lane 1: unpurified recombinant DSsAOMT5; Lane 2: purified recombinant DSsAOMT5; Lane 3:purified recombinant TSsAOMT5.**
